# The Benefits of Using Active Remote Patient Management for Enhanced Heart Failure Outcomes in Rural Cardiology Practice: Single-Site Retrospective Cohort Study

**DOI:** 10.2196/49710

**Published:** 2024-11-26

**Authors:** William Craig, Suzanne Ohlmann

**Affiliations:** 1 Craig Cardiovascular Center Seguin, TX United States; 2 Craig Cardiovascular Center San Antonio, TX United States

**Keywords:** rural, remote patient monitoring, heart failure, heart failure hospitalizations, office visits, rural health inequalities, telehealth

## Abstract

**Background:**

Rural populations have a disproportionate burden of heart failure (HF) morbidity and mortality, associated with socioeconomic and racial inequities. Multiple randomized controlled trials of remote patient monitoring (RPM) using both direct patient contact and device-based monitoring have been conducted to assess improvement in HF outcomes, with mixed results.

**Objective:**

We aimed to assess whether a novel digital health care platform designed to proactively assess and manage patients with HF improved patient outcomes by preventing HF re-exacerbations, thus reducing emergency room visits and HF hospitalizations.

**Methods:**

This was a single-site, retrospective cohort study using electronic medical record (EMR) data gathered from 2 years prior to RPM initiation and 2 years afterward. In January 2017, this single center began enrolling New York Heart Association (NYHA) class II and class III patients with HF prone to HF exacerbation into an RPM program using the Cordella HF system. By July 2022, 93 total patients had been enrolled in RPM. Of these patients, 87% lived in rural areas. This retrospective review included 40 of the 93 patients enrolled in RPM. These 40 were selected because they had 2 years of established EMR data prior to initiation of RPM and 2 years of post-RPM data; each consented to this Sterling IRB–approved study.

**Results:**

We included 40 patients with at least 4 years of follow-up, including 2 years prior to RPM initiation and 2 years after RPM initiation. In the 2 years after RPM initiation, check-up calls increased 519%, medication change calls increased 519%, and total calls increased by 519%. Emergency room visits for HF fell 93%, heart failure hospitalizations fell 83%, and all other cardiovascular hospitalizations fell 50%. Additionally, the total number of office visits declined by 15% after RPM, and unscheduled or urgent office visits declined by 73%.

**Conclusions:**

Daily monitoring of trends in vital sign data between engaged patients and a collaborative team of clinicians, incorporated into daily clinical workflow, enhanced patient interactions and allowed timely response or intervention when HF decompensation occurred, resulting in a reduction of outpatient and inpatient clinical use over more than 2 years of follow-up.

## Introduction

Rural populations have a disproportionate burden of disease, particularly obesity, diabetes, smoking, hypertension, and coronary heart disease, all of which are associated with poor health outcomes [1,2]. This is specifically reflected in the fact that rural populations have worse outcomes for heart failure (HF) morbidity and mortality and is compounded by racial and socioeconomic inequities [3-5]. Rural patients also receive less outpatient cardiac management and are more likely to present to emergency care or be hospitalized in the first year of HF diagnosis compared to urban patients [6]. Remote patient monitoring (RPM) of patients with HF to reduce HF hospitalizations and mortality has been studied in multiple randomized clinical trials, with mixed results [7-11]. These studies used a variety of RPM systems for HF management that included some combination of structured telephone calls and daily weight, blood pressure, heart rate, and symptom questionnaires. RPM trial meta-analyses have failed to show any consistent benefit from RPM when compared with the usual standard of care [12-16]. Many of the studies may not have used optimal monitoring equipment or were unable to incorporate appropriate, efficient clinical workflows into the process of RPM. Patient compliance may have also been negatively impacted by using automated telephone menus for patient data reporting rather than direct, clinician-to-patient communication for feedback, medication adjustment, and monitoring of patient acceptance and engagement in treatment modalities. Here, we report results of a single-center study on the use of a novel RPM system in a rural population with HF. We hypothesize that active patient management with RPM, when integrated into a standard cardiology workflow, enhances patient care, improves patient satisfaction, and reduces emergency room (ER) visits and HF hospitalizations.

## Methods

### Overview

This is a retrospective cohort review using electronic medical record (EMR) data from a group of New York Heart Association (NYHA) class II and III patients with HF who were followed over a 4-year period at a single institution, including 2 years prior to and 2 years following the initiation of a novel digital system to assess the impact of RPM on HF management.

Between January 2017 and July 2022, this single site began enrolling patients with HF who showed demonstrated difficulty maintaining clinical HF compensation into the Cordella HF System (Endotronix Inc) program. Over that period, a total of 93 patients were enrolled, 87% of whom lived in rural areas (defined as more than 1 hour from the city limits of an urban center with tertiary care).

Of the 93 total patients in this study, we included 40 who had both 2 years of pre-RPM EMR data established within the practice and 2 years of post-RPM EMR data. We excluded 53 of the 93 total patients who had been enrolled in Cordella due to the absence of sufficient data: either they lacked 2 years of established EMR data prior to joining the RPM program, or they had not accrued the full 2 years of data after initiation at the time of this review. Additionally, 3 patients in the excluded group dropped out of the RPM program prior to completing 2 years of enrollment, and 4 patients died prior to completion of 2 years of RPM. Of note, of the 4 deceased patients, 2 would not have had sufficient EMR data within the practice prior to enrolling in RPM.

**Figure 1:**
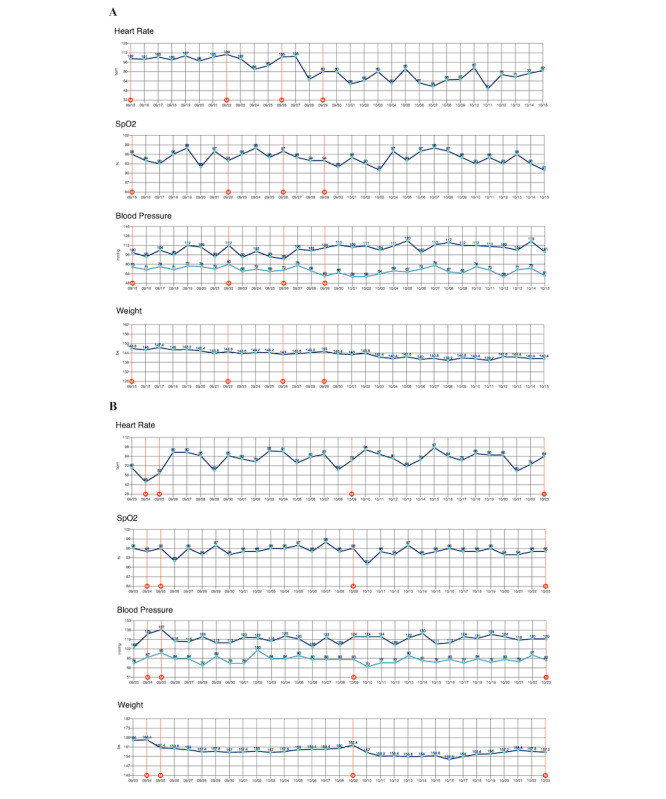
Examples of 2 clinical case applications of the Cordella Remote Patient Monitoring System in outpatients with heart failure at a single heart failure clinic lasting 2 years. (A) Vital signs over time (blue horizontal lines) and heart failure medication changes (orange vertical lines) for a case in which the system was used to lower heart rate. (B) Vital signs over time and diuretic medication changes for a case that used the system to reduce elevated weight indicative of edema. For a higher-resolution version of this figure, see Multimedia Appendix 1.

The Cordella HF System is a novel, free-standing, digital health care platform designed specifically to proactively manage outpatients with HF and the multiple factors that lead to HF re-exacerbation and readmission. The system uses a wireless/cellular tablet computer to receive data from multiple Bluetooth-enabled peripherals to assess vital signs. The tablet computer, weight scale, blood pressure cuff, and pulse oximeter were provided to patients for home use with online and telephone technical support. In this mostly rural population, even older patients with limited technical experience were able to follow telephone instructions to set up the equipment in their homes. Patients were instructed to use these devices daily and to answer questions on their current clinical status via their tablets. The data were then securely transmitted to an online clinician portal and reviewed remotely from the physician’s office as either tabular or trend data (Figure 1).

Importantly, the workflow for daily RPM for these patients was incorporated into usual clinical practices. The same interdisciplinary team reviewed the daily trends for each patient as part of an integrated workflow in the rural practice. A full-time clinic nurse was dedicated to assisting review of the patients’ data and interacting with the patients by telephone and the tablet messaging system as often as necessary. The cardiologist and nurse discussed problems identified in the daily trends, and the nurse then contacted patients if additional information was needed. When a lifestyle or medication intervention became necessary, the team contacted the patient to make that change. The patient’s response was then monitored on subsequent daily RPM readings with further adjustments made as needed.

All discussions between the cardiologist and the nurse were documented within the Cordella Patient Management Portal (PMP). Telephone calls to patients to discuss their status and recommend medication and lifestyle modifications were also documented in the PMP. Medication changes were annotated on the trend plots to mark the response. Permanent medication changes were documented in the patient’s EMR, as the PMP is not currently integrated with the EMR. The contact time spent in daily review of patient data, discussion of changes noted in data trends, and interaction with the patient for further information or medication change was synchronously captured by the PMP. All patients were contacted at least monthly to discuss their clinical status, note visits with their other physicians, document ER visits or hospitalizations, and ensure that no changes had been made to pertinent cardiac medications.

All cardiovascular hospitalizations, ER visits, and HF hospitalizations were assessed in the 2-year period prior to initiation of RPM and the 2-year period following RPM initiation. Similarly, the total number of telephone calls made monthly to patients by the clinical team, noting the reason for the calls, was assessed in the 2-year period prior to initiation of RPM and the 2-year period following RPM initiation, as well as any unplanned clinic visits. Data on each end point were compared for the period before and after the initiation of RPM using Wilcoxon signed-rank tests. Patient compliance, defined as data transmission to the portal 5 of 7 days per week, and clinician compliance, defined as clinicians checking the PMP daily, were also assessed.

### Ethical Considerations

This study was a retrospective cohort analysis of electronic patient data accrued over a 4-year period. The study received institutional review board approval (Sterling IRB; 8276-WECraig), and each participant signed informed consent documentation prior to having their data collected for daily Cordella review, as well as for the purposes of this study. Participant privacy and anonymity were achieved through elimination of any patient identifiers such as name or dates of birth, which were replaced by an anonymous, numeric system for data tabulation and analyses. The participants did not receive compensation for their participation in the study.

## Results

This study included 40 patients with at least 4 years of follow-up, including 2 years prior to RPM initiation and 2 years following RPM initiation. The patients had a mean age of 76.9 (SD 9.5) years. A majority were female (n=23, 58%) and had a high comorbidity burden, including 40% (n=16) with chronic kidney disease, 55% (n=22) with atrial fibrillation, 15% (n=6) with aortic stenosis, and 68% (n=27) with mitral regurgitation. Most patients were NYHA class III (n=32, 80%) and 42% (n=17) had an ejection fraction ≥50%.

In the 2 years prior to RPM initiation, the average number of check-up calls per month to all 40 patients was 7.5 (SD 0.1), and the average number of medication calls per month to all 40 patients was 4.3 (SD 0.2), with a total average number of calls per month numbering 11.8 (SD 0.3). In the 2 years that followed RPM initiation, check-up calls increased 519% (to 46.4, SD 0.8 per month; *P*<.001), medication change calls increased 519% (to 26.6, SD 0.7 per month; *P*<.001), and total calls increased 519% (to 73, SD 1.3 per month; *P*<.001).

In the 2 years prior to RPM initiation, there were 48 unplanned office visits, 15 ER visits for HF, and 36 HF hospitalizations; all other cardiovascular hospitalizations numbered 38. In the 2 years that followed RPM initiation, ER visits for HF fell 93% (to 1 visit; *P*=.006), HF hospitalizations fell 83% (to 6 visits; *P*=.003), all other cardiovascular hospitalizations fell 50% (to 19 visits; *P*=.06), and unplanned office visits fell 73% (to 13 visits, *P*=.001) (Table 1). Patient compliance was 88% at 6 months, 90% at 1 year, and 95% at 2 years. Clinician compliance was 100% throughout the study period.

**Table 1:** Monthly calls and total visits in the 24 months before remote patient monitoring (RPM) and 24 months after RPM.

Call/visit type		24 months before RPM	24 months after RPM	Percentage change		*P* value	
**Monthly calls, mean (SD)**							
	Medication change	4.3 (0.2)	26.6 (0.7)		+519%	<.001	
	Check-up	7.5 (0.1	46.4 (0.8)		+519%	<.001	
	Total	11.8 (0.3)	73 (1.3)		+519%	<.001	
**Total visits, n**							
	Unplanned office visit	48	13		–73%	.001	
	ER^a^ visit for HF^b^	15	1		–93%	.006	
	HF hospitalization	36	6		–83%	.003	
	All other CV^c^ visits	38	19		–50%	.06	

^a^ER: emergency room.

^b^HF: heart failure.

^c^CV: cardiovascular.

## Discussion

### Principal Findings

This retrospective study shows a dramatic decrease in hospitalizations, ER visits, and urgent office visits after active management with daily RPM was integrated into standard cardiology care delivery. This management approach used daily RPM in conjunction with frequent patient contact and timely adjustment of medication and lifestyle issues. Management of patients with advanced HF is a dynamic process. Each patient’s unique comorbidities, diet, and lifestyle contribute to decompensations between regularly scheduled office visits. Swift identification and treatment of the precursors to decompensation, a hallmark of this study’s success, significantly reduced hospitalizations, ER visits, and urgent office visits.

This study identified multiple factors that contributed to the successful reduction in HF decompensations. Most relevant, daily RPM using this model facilitates frequent clinician-patient interaction and care adjustments. Monitoring trend plot flow sheets combined with patient symptom reporting reduces clinician apprehension when making care adjustments remotely.

Patient response to medication changes was assessed quickly via vital sign trends, telephone calls, and further medication adjustment, decreasing the need for in-office follow-up.

Equally important are the challenges of patient engagement and education, both of which contribute to the success of decreasing HF decompensations. Frequent interaction with the cardiologist and nurse team facilitated numerous discussions of fluid and sodium intake, better understanding of the complex nature of HF disease and treatment and the benefits of monitoring data trends through the shared portal, and increased adherence to the current medication regimen.

Close monitoring with RPM and frequent patient interactions within this rural setting, reflected by the increase in telephone calls, improved patients’ clinical well-being and was associated with a dramatic reduction in ER visits, HF hospitalizations, and unplanned office visits.

The Cordella HF system allowed patients to observe and track their own health data, provided the foundation for patient education, and facilitated shared decision-making with the clinical team. This connection between clinician and patient via shared data with frequent, personalized clinical interventions facilitated patient education and led to the observed increase in patient reporting and compliance over time.

The role of a dedicated nurse for RPM is an equally important factor in the success of this study. The HF nurse monitors the daily patient data and calls patients to assess symptoms and explain medication changes. The role evolved to include coaching for support, education, and feedback regarding the multiple factors involved in HF management. Over time, the HF nurse mediated in both clinical and lifestyle issues pertinent to HF management, providing both encouragement and intervention when needed and reassurance when the patient was stable. While this study included 40 patients, our practice now uses this system for more than 70 patients with no further disruption in clinic workflow and no additional staff, a positive indicator of the scalability of this technology.

The workflow used for RPM in the study was easily incorporated into a standard outpatient cardiology practice. The managing cardiologist maintained a busy daily clinic schedule and hospital rounds. The dedicated HF nurse was employed by the practice, and for patients eligible for chronic care management (CCM), CCM billing not only covered the cost of the Cordella HF system and the dedicated nurse but was also cost-effective, generating revenue for the practice.

### Limitations and Future Research

The major limitation of the study is its retrospective nature. However, all patients with a full 24 months of RPM were included. Those patients who had enrolled for monitoring but dropped out prior to completing 24 months, or who had not completed 24 months of monitoring at the time of data analysis, were excluded. A second of the study’s limitations lies in the intense nature of monitoring and frequent interaction with patients. Due to the relatively small cohort of patients, this intensive effort was feasible within the rural cardiology practice, which mirrors many of those delivering care in the rural setting. It remains to be seen if this same workflow could be expanded to a larger clinical population with HF.

Further exploration into reimbursement is merited—for both Medicare and non-Medicare patients—as well as a cost-benefit analysis to include projected savings due to the decrease in ER visits and HF hospitalizations. This workflow also offers the opportunity to explore specific combinations of data and alerts that can improve early identification of changes in status. This information may also enhance HF management by expanding the multidisciplinary team caring for these patients.

### Conclusion

Active HF management with RPM and a focused multidisciplinary team reduced ER visits for HF by 93%, HF hospitalizations by 83%, and unplanned office visits by 73% in this retrospective study. Daily monitoring of trends in vital sign data between engaged patients and clinicians, when incorporated into the daily clinical workflow, allowed for immediate response to the precursors of HF decompensation and intervention. The success of this study’s proactive model of HF management using the Cordella HF system stands in great contrast to the current reactive model of HF urgent office visits, ER visits, and HF hospitalizations.

## Appendix

Multimedia Appendix 1 Examples of 2 clinical case applications of the Cordella Remote Patient Monitoring System in outpatients with heart failure at a single heart failure clinic lasting 2 years. (A) Vital signs over time (blue horizontal lines) and heart failure medication changes (orange vertical lines) for a case in which the system was used to lower heart rate. (B) Vital signs over time and diuretic medication changes for a case that used the system to reduce elevated weight indicative of edema.

## Abbreviations

CCM: chronic care management
CV: cardiovascular
EMR: electronic medical record
ER: emergency room
HF: heart failure
IRB: institutional review board
NYHA: New York Heart Association
PMP: Patient Management Portal
RPM: remote patient monitoring

## Data Availability

All data generated or analyzed during this study are included in this published article.
