# Supplementary figures and images for: The Benefits of Using Active Remote Patient Management for Enhanced Heart Failure Outcomes in Rural Cardiology Practice: Single-Site Retrospective Cohort Study
Source: J Med Internet Res. 2024 Nov 26;26:e49710. doi: 10.2196/49710 (PMC11632278; doi:10.2196/49710)

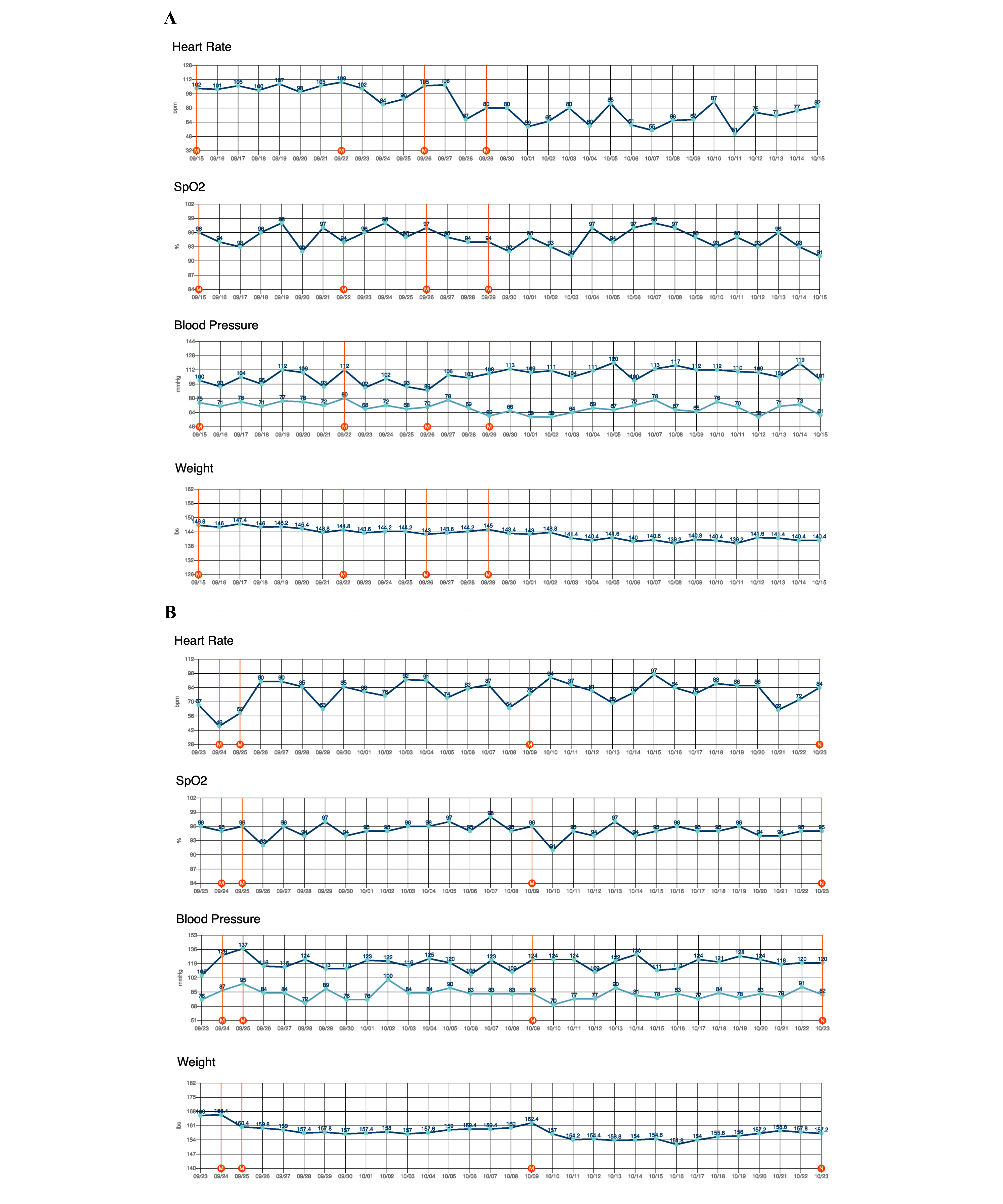

Supplement: Multimedia Appendix 1 [file jmir_v26i1e49710_app1.png]
